# Chronic Lead Toxicity in a Family: A Case Report

**DOI:** 10.7759/cureus.66574

**Published:** 2024-08-10

**Authors:** Pooja Sadhu, Sampada Tambolkar, Devika Jadhav, Avinash Daru

**Affiliations:** 1 Department of Pediatrics, Dr. D. Y. Patil Medical College, Hospital & Research Centre, Dr. D. Y. Patil Vidyapeeth (Deemed to be University), Pune, IND

**Keywords:** chelation therapy, blood lead levels, neurotoxicity, occupational health, lead toxicity

## Abstract

Lead poisoning, also known as plumbism, is a significant global health concern, occurring more commonly in the pediatric age group. The widespread use of lead in developing and developed countries due to industrialization has led to the contamination of the environment and lead toxicity. With the increasing number of cases, it is very important to identify and treat lead toxicity at the earliest to prevent detrimental side effects like neurocognitive impairment, developmental regression, coma, and death. This case report depicts a family whose parents are employed in the battery recycling sector, putting them and their children at risk for lead poisoning.

## Introduction

Lead is a naturally occurring, non-biodegradable heavy metal. Lead poisoning in children occurs mainly due to contaminated soil, dust, lead-based paint chippings, and occupational exposure from adults to their children. It is commonly seen in battery manufacturing and recycling, lead mining, construction workers, and lead smelting [1]. There is no safe level of exposure to lead, as it can cause deleterious effects even at the lowest blood lead concentrations [2]. Factors like age, nutritional status, period of exposure, and route of exposure contribute to the levels of toxicity [3].

Lead poisoning is widespread and is seen in almost one-third of the pediatric population. Globally, 800 million children have blood lead levels of 5 micrograms or higher, of which 275 million are from India, and 64 million of them have a blood lead level of more than 10 micrograms [4].

## Case presentation

A three-year-old male child, fourth born, out of a non-consanguineous marriage with both parents working in the battery recycling industry, presented with one episode of unprovoked seizure and a blank staring look towards the left side. It was associated with tightening of bilateral upper and lower limbs without any deviation of mouth. This episode lasted for 10-15 minutes until the child was brought to the hospital where it was aborted with one dose of injection midazolam. The parents reported that the child had been experiencing vomiting and abdominal distension for one week. Additionally, they mentioned complaints of constipation and pica (an eating disorder) for the past two months.

Family history revealed that the elder sibling, an eight-year-old female, third born, has a history of seizures. The first episode occurred at five months of age and was characterized by up-rolling of eyes and tonic-clonic posturing of all four limbs. She also started developing difficulty in standing up and walking, associated with wobbling of legs at 1.5 years of age, and had atrophy of calf muscles and contractures at ankle joints. She had multiple episodes of seizures since then and was started on multiple anti-seizure medications. A history of pica was also reported. The sibling was evaluated for these seizures. The electroencephalography (EEG) results were normal, but magnetic resonance imaging (MRI) of the brain indicated unilateral brain volume loss. Occupational history revealed about parents working in the automobile battery recycling industry, from where the remnants and chemicals were taken home, resulting in exposure to their children too.

**Figure 1:**
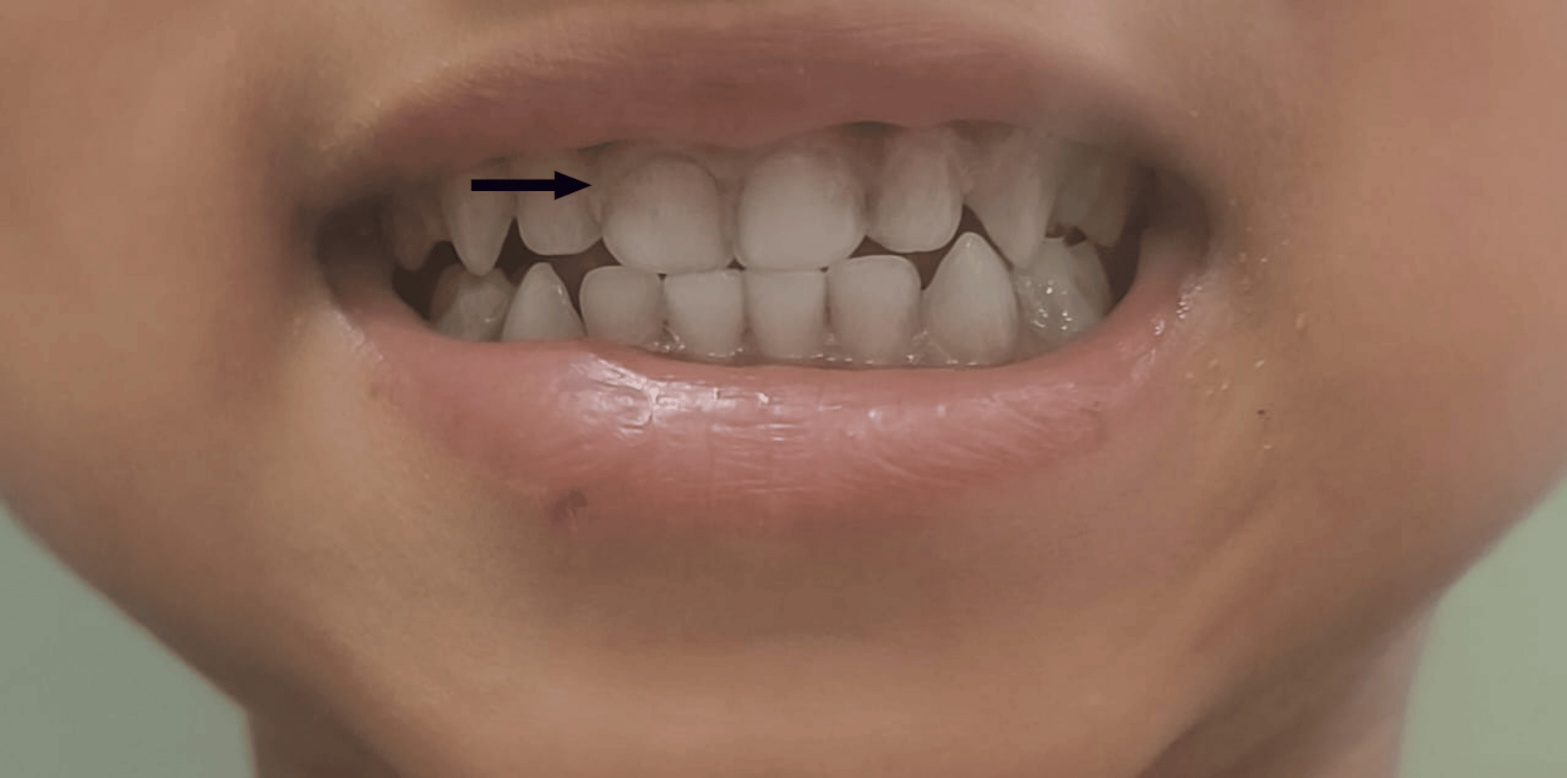
Burton's lines on the gums of the patient

The patient on admission was cardiorespiratory stable, and irritable, with the Glasgow Coma Scale (GCS) score of 9/15, with bilateral pupils equal and reacting to light. On examination, the patient had pallor and bluish-purple discoloration (Burton's lines) on the gums (Figure 1). 

**Figure 2:**
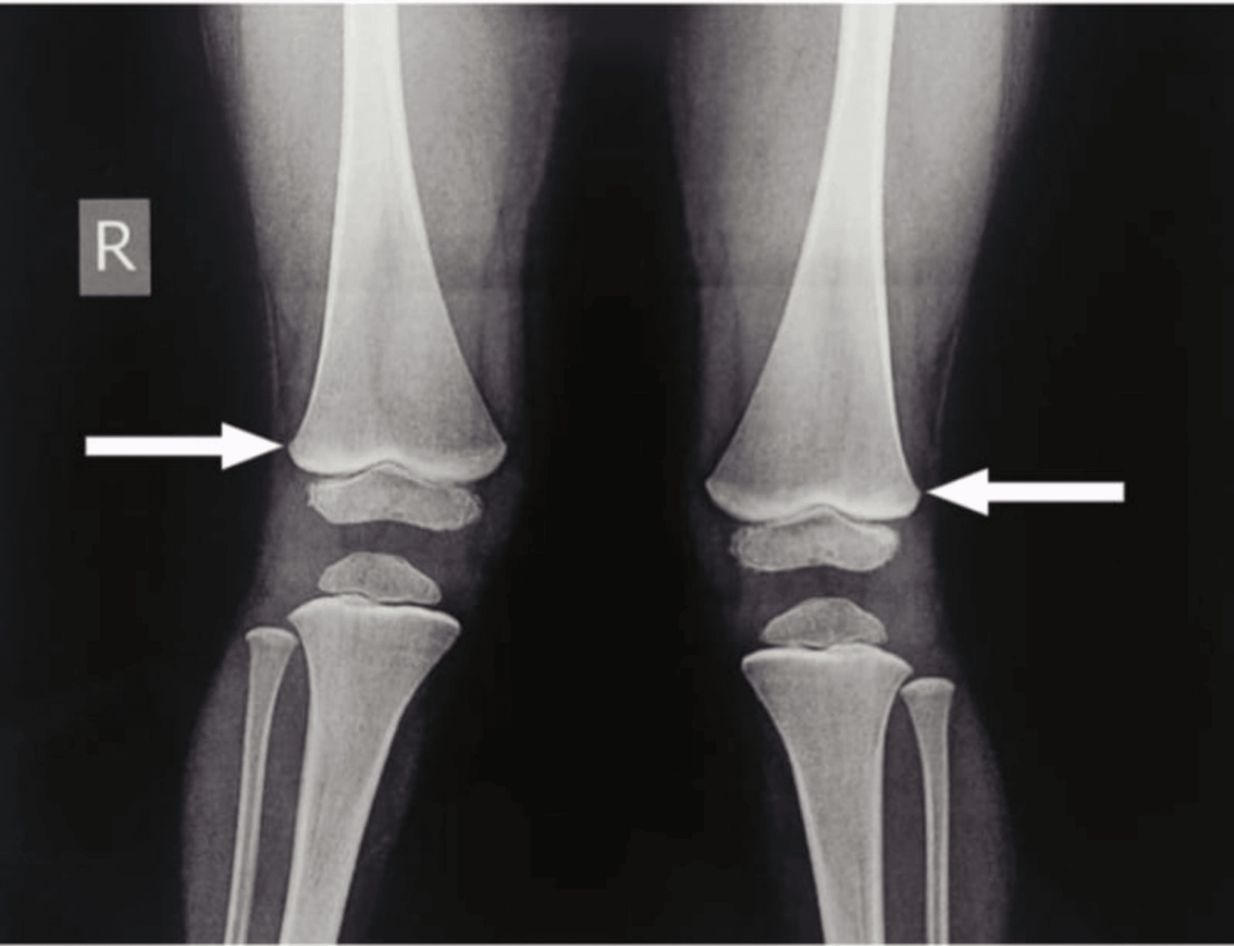
Lead lines at the metaphysis of bilateral long bones

At presentation, the systemic examination was normal. Radiological investigations revealed normal ultrasonography (USG) of the abdomen and pelvis. The EEG revealed a normal EEG during the sleep state. The MRI of the patient showed a small subcentimeter-sized cerebrospinal fluid (CSF) intensity cyst in the body region of the anterior corpus callosum, suggestive of a small corpus callosal cyst; no other neuro-parenchymal abnormality was noted. The radiograph of long bones revealed lines of increased density (lead lines) at the metaphysis of both femurs (Figure 2). 

Biochemical investigations of the patient were normal, except for low hemoglobin levels (8.9 g/dl) (Table 1). The peripheral blood smear was suggestive of hemolysis and few reactive lymphocytes without basophilic stippling. The hemolytic workup was negative.

**Table 1:** Biochemical investigations of the patient MCV: mean corpuscular volume; MCH: mean corpuscular hemoglobin; MCHC: mean corpuscular hemoglobin concentration; SGOT: serum glutamic-oxaloacetic transaminase; SGPT: serum glutamic-pyruvic transaminase; ALP: alkaline phosphatase; GGT: gamma-glutamyl transpeptidase; DCT: direct Coombs test; G6PD: glucose-6-phosphate dehydrogenase

Investigation	Value	Reference range
Hemoglobin	8.9	11-14 g/dl
WBC	17500	5000-12000/µL
Platelet count	350000	150000-410000/µL
Hematocrit	28.4	33-42%
MCV	78.9	74-89 fL
MCH	24.6	24-30 pgms
MCHC	31.10	31-37 g/dl
Calcium	8.60	8.8-10.8 mg/dl
Magnesium	1.90	1.80-2.40 mg/dl
Phosphorus	6.30	2.6-4.7 mg/dl
Sodium	137	138-145 mmol/ltr
Potassium	3.51	3.5-5.1 mmol/ltr
Chloride	105	98-107 mmol/ltr
Serum ferritin	85.77	21.81-274.66 ng/dl
Total bilirubin	0.27	0.22-1.20 mg/dl
Bilirubin conjugated	0.15	<0.5 mg/dl
Bilirubin unconjugated	0.12	0.1-1 mg/dl
SGOT	64	8-60 U/Lt
SGPT	30	7-55 U/Lt
ALP	163	142-335 U/Lt
GGT	15	<21 U/L
Urea	24	17-49 mg/dl
Creatinine	0.40	0.19-0.49 mg/dl
Reticulocyte count	11.20	0.5-2.5%
DCT	Negative	Negative
G6PD	9.15	4.6-13.5
Sickling test	Negative	Negative

In view of the suspicion of lead poisoning, blood lead levels were advised. The patient's blood lead level was 55.57 (normal: <3.5 mcg/dl). Considering the symptoms of the elder sibling, chemical exposure, and lead toxicity within the family, the mother and elder sibling were also screened for blood lead levels, which were >65 and 42.81 mcg/dl, respectively (Table 2).

**Table 2:** Blood lead levels of the family

	Blood lead levels	Reference range
Patient	55.57 mcg/dl	<3.5 mcg/dl
Sibling	>65 mcg/dl	<5 mcg/dl
Mother	42.81 mcg/dl	<5 mcg/dl

The patient, as well as the mother and the sibling, was started on D-penicillamine, and all of them have been advised to follow up for the monitoring of blood lead levels, complete blood counts, and renal function tests. They have been counseled regarding the prevention of further exposure, and all other family members have been advised to get screened for blood lead levels at the earliest.

## Discussion

Lead is absorbed through the lungs by inhalation or by the accidental consumption of contaminated water or food through the gastrointestinal tract or through the skin. This lead is distributed in the blood, bones, and tissues. In the blood, 99% of lead is stored in the red blood cells and 1% in the plasma, which is more harmful. Ninety-five percent of lead is stored in the bones which can remain there for decades. Lead is also known to cross into the fetus through the placenta [5].

Lead toxicity leads to multiple systemic involvement, mainly by free radical damage. Many cell signaling pathways mediated by calcium and zinc are affected by lead as they all are divalent cations [6].

Stomach pain, loss of appetite, and anemia can occur at low blood lead levels and these symptoms lack diagnostic value, but it is significant to identify, due to the lasting neurotoxic effects. Severe lead poisoning leads to symptoms like lethargy, abdominal cramps, anorexia, and irritability. Gradually vomiting, clumsiness, and ataxia set in. Hyperirritability, stupor, seizures, and coma can also occur. These symptoms are seen at blood lead levels of 50-70 mcg/dl [7].

Microcytic anemia, seen in lead poisoning, is due to the inhibition of porphobilinogen synthase and ferrochelatase, which prevents porphobilinogen formation and the incorporation of iron into protoporphyrin IX leading to ineffective heme synthesis [1]. Basophilic stippling is seen due to the inhibition of pyrimidine 5'-nucleotidase [8].

Other features include renal proximal collecting tube dysfunction, deposition of urate crystals in joints known as saturnine gout, hypertension, impaired thyroid function, and gastrointestinal symptoms like abdominal pain, constipation, and anorexia [8]. Signs of lead poisoning include bluish-purple lines, caused by a reaction between lead and sulfur ions released by the oral bacterial activity known as Burton's lines [9], and dense metaphyseal bands seen on long bone radiographs due to increased calcium deposition known as lead lines [10].

Treatment options are chelation therapy with dimercaprol or British anti-Lewisite (BAL), dimercaptosuccinic acid (DMSA), edetate calcium disodium (versenate), and D-penicillamine. Chelation should always be considered if blood lead levels are more than 45 mcg/dl. Dietary supplementation of iron, zinc, calcium magnesium, vitamin C, and vitamin D helps in the prevention of excessive absorption of lead [11].

## Conclusions

Lead poisoning is a preventable occupational disease that occurs in children of parents working in the lead smelting and battery recycling industries or from direct exposure to lead-based dust, paints, or contaminated drinking water. Identifying the source and prevention of exposure to these agents is the only way to prevent lead poisoning. Even at low blood lead levels, it is known to cause cognitive and neurological deficits and a significant impact on the developing brains of these children. Early diagnosis and management are important to prevent severe lead poisoning which can lead to convulsions, coma, and death. Regular follow-ups with blood lead levels and checking the adherence to treatment along with counseling for the further prevention of exposure are of significance.
